# Spontaneous Coronary Artery Dissection (SCAD): Unveiling the Enigma of the Unexpected Coronary Event

**DOI:** 10.1007/s11883-025-01328-5

**Published:** 2025-08-15

**Authors:** Cristiane C. Singulane, Shuo Wang, Kelsey Watts, Macy E. Stahl, LeAnn Denlinger, Rachel Lloyd, Pranavi Pallinti, Lauren Preston, Mohamed Morsy, Odayme Quesada, Angela Taylor, Randy K. Ramcharitar, Mete Civelek, Patricia F. Rodriguez-Lozano

**Affiliations:** 1 Department of Medicine, Cardiovascular Division, University of Virginia Health, Charlottesville, VA USA; 2 Department of Genome Sciences, University of Virginia, Charlottesville, VA USA; 3 Department of Kinesiology, University of Virginia, Charlottesville, VA USA; 4 School of Medicine, University of Virginia, Charlottesville, VA USA; 5 Oakland University William Beaumont School of Medicine, Rochester, MI USA; 6 Division of Cardiovascular Health and Disease, University of Cincinnati Medical Center, Cincinnati, OH USA; 7 Women’s Heart Center, The Christ Hospital Heart and Vascular Institute, Cincinnati, OH USA; 8 Department of Radiology and Medical Imaging, University of Virginia Health, 1215 Lee St, Box 800158, Charlottesville, VA 22908 USA

**Keywords:** Spontaneous coronary artery dissection, Precision medicine, Multiomics, Social determinants of health

## Abstract

**Purpose of Review:**

Spontaneous coronary artery dissection (SCAD) is an underrecognized cause of acute coronary syndrome, primarily affecting younger women without traditional risk factors. This review synthesizes current knowledge, identifies research gaps, and explores how precision medicine can improve diagnosis, treatment, and patient outcomes.

**Recent Findings:**

SCAD is clinically and biologically heterogeneous, with variation in presentation, etiology, and recurrence risk. Contributing factors include sex-specific hormonal differences, vascular abnormalities, genetic predisposition, psychosocial stressors, and social determinants of health. Despite growing awareness, major gaps persist in risk stratification and tailored care. Emerging tools, including genomics, radiomics, and multiomics, offer opportunities to define SCAD subtypes and personalized management.

**Summary:**

A precision medicine framework integrating molecular, imaging, and social data may transform SCAD care. Continued research is essential to improve early detection, optimize therapy, and reduce recurrence.

## Introduction

Spontaneous coronary artery dissection (SCAD) is an underrecognized cause of acute coronary syndrome (ACS), disproportionately affecting young to middle-aged women with few typical cardiovascular risks. It involves a non-atherosclerotic, non-traumatic, non-iatrogenic tear or intramural hematoma within the coronary artery wall, causing coronary artery dissection with acute development of a false lumen and subsequent obstruction of coronary blood flow [1, 2]. This can result in significant myocardial ischemia, infarction, and, in rare instances, sudden cardiac death. SCAD’s clinical course is often complicated by high recurrence rates, persistent post-event symptoms, and substantial psychosocial impact [3–5]. Standard ACS management protocols may be inappropriate or even harmful in this context, underscoring the need for more tailored diagnostic, risk stratification, and therapeutic strategies.

Precision medicine offers a promising pathway to address these challenges through integration of multi-dimensional data – including genetic, environmental exposures, imaging and psychosocial data to better define SCAD phenotypes. Tools such as multiomics, radiomics, and advanced imaging may help uncover distinct molecular mechanisms, identify novel biomarkers, and enable individualized treatment approaches [6, 7].

This review will summarize the current knowledge of SCAD, highlight key research gaps, and explore the potential for incorporating a precision medicine framework to advance diagnosis, management, and long-term outcomes in this unique and complex patient population.

## Current Knowledge and Perspectives on SCAD

### Epidemiology and Risk Factors

SCAD is increasingly recognized as a significant cause of ACS. Although true prevalence remains uncertain due to diagnostic challenges, SCAD is estimated to account for up to 5% of ACS cases, and up to 35% in women under age 50 presenting with MI [8, 9]. It predominantly affects women (~ 90%), often in their 40–50 s [3, 10], with low burden of traditional cardiovascular risk factors, with hypertension being the most common (approximately 30% of cases) [10, 11]. In contrast, nontraditional factors such as emotional or physical stress and neuropsychiatric conditions—including anxiety, depression, and migraine—are frequently reported in SCAD populations [3, 10].

Pregnancy-associated SCAD (P-SCAD) accounts for < 5–17% [4] of cases and is the leading cause of pregnancy-related MI [4, 12, 13]. It is associated with peripartum risk factors, including multiparity, preeclampsia, and fertility treatment [1, 2, 14]. Importantly, P-SCAD is an independent predictor of major adverse cardiovascular events (MACE), highlighting the need for long-term surveillance and individualized care in this population [3].

Importantly, while men are less frequently affected, they tend to present at a younger age, with a median age of approximately 49 years, compared to 52 years in women. Physical exertion, particularly isometric exertion, is more commonly reported as a trigger in men (58–72%); in contrast, emotional stress is more frequently reported in women (55–59%) [15, 16]. Men are less likely to have atypical chest pain or to have recurrent emergency department visits for chest pain, with reported rates of 11% compared to 25% in women during long-term follow-up [15]. However, sex-specific differences in long-term outcomes remain poorly characterized due to underrepresentation of men in most SCAD cohorts.

Most published SCAD data derive from predominantly white North American populations, raising concerns about underdiagnosis or underreporting in Black, Hispanic, Indigenous, and Asian individuals [3, 4, 9, 10, 17]. This limits our understanding of potential racial and ethnic variation in disease susceptibility and outcomes.

Among non-atherosclerotic arteriopathies, fibromuscular dysplasia (FMD) has emerged as the most consistently associated condition with SCAD. A strong association exists between the two, with non-coronary vascular abnormalities identified in up to 56.4% of patients who undergo comprehensive vascular screening [3]. The renal, iliac, and cerebrovascular arteries are most frequently affected [3, 18]. Although the exact pathophysiologic link remains unclear, this relationship highlights the importance of routine vascular imaging after a SCAD diagnosis to identify potential systemic arteriopathies and inform risk assessment.

Beyond acquired risk factors and associated arteriopathies, inherited conditions have been investigated as potential contributors to SCAD susceptibility. While monogenic inheritance is uncommon, familial clustering has been described; however, true familial SCAD appears to account for fewer than 1% of cases, and large multigenerational pedigrees have not been reported, suggesting a predominantly polygenic risk profile [5, 19, 20]. The role of rare inherited connective tissue disorders—such as Marfan syndrome, vascular Ehlers-Danlos syndrome, and Loeys-Dietz syndrome—appears limited to a small subset of patients [3, 5, 21]. Autoimmune diseases are similarly infrequent (< 5%), and current evidence does not support a direct causal relationship with SCAD [3, 5].

### Pathophysiology

The pathophysiology of SCAD centers around two primary mechanisms of arterial wall disruption. The “inside-out” model involves an intimal tear that allows blood to enter a false lumen, while the “outside-in” model describes hemorrhage from the vasa vasorum into the media, forming an intramural hematoma that compresses the true lumen [22, 23]. Optical coherence tomography (OCT) and autopsy studies increasingly support the outside-in mechanism, particularly in cases without visible intimal disruption [24, 25]. Findings from OCT suggest that arterial wall hemorrhage, rather than true intimal dissection, is the predominant initiating mechanism in most SCAD patients, given the frequent absence of identifiable fenestrations [3, 25]. This observation has important therapeutic implications, especially regarding the use of dual antiplatelet therapy (DAPT) in patients managed conservatively without revascularization [11]. In many patients, hematoma expansion may lead to secondary tearing, further complicating efforts to distinguish the initial event on angiography.

While the initial SCAD event is not considered an inflammatory process, increasing evidence suggests that an exaggerated inflammatory response post-SCAD may contribute to prolonged symptoms, recurrent events, and disease progression. Emerging data suggest that SCAD patients exhibit a heightened inflammatory response; for instance, histologic and immunohistochemical analyses reveal increased inflammatory cell infiltration in dissected arterial segments in patients with delayed death compared to those with sudden death [23, 24]. These findings suggest that inflammation is more likely a secondary phenomenon than a primary trigger. Furthermore, one study observed higher systemic inflammatory markers (e.g., C-reactive protein (CRP), interleukin-6 (IL-6), and neutrophil-to-lymphocyte ratio [NLR]) in SCAD patients compared to those with atherosclerotic ACS [26]. This suggests a global inflammatory process that could serve as both a biomarker of disease activity and a potential therapeutic target.

The striking female predominance suggests a sex-specific biological vulnerability, potentially involving hormone-mediated modulation of vascular tone, extracellular matrix integrity, and autonomic regulation. Estrogen is known to influence vascular tone, endothelial function, and extracellular matrix remodeling through effects on nitric oxide, prostacyclin, and matrix metalloproteinases [27–29]. SCAD frequently occurs during periods of hormonal fluctuation—such as perimenopause or postpartum—suggesting hormonal shifts may unmask underlying arterial vulnerability. However, direct mechanistic links between circulating hormone levels and SCAD remain unproven [4, 5, 30].

This hormonal susceptibility may be further amplified in the context of pregnancy, where a convergence of vascular, hormonal, and hemodynamic stressors likely contributes to an elevated risk of dissection. P-SCAD may involve additional vascular stressors. Proposed mechanisms include placental hormone- or growth factor–induced weakening of the arterial media via reduced collagen synthesis [23, 28, 31], alongside increased hemodynamic load and impaired endothelial function during late pregnancy and postpartum [23, 28, 32]. These hypotheses remain under investigation.

### Clinical Presentation

SCAD most commonly presents as an acute myocardial infarction with non-ST-elevated myocardial infarction (NSTEMI) being the most common clinical presentation [10, 11]. In a large Mayo Clinic Multicenter SCAD Registry, comprised of 1196 SCAD subjects, 58% had NSTEMI, 39% had ST-elevation myocardial infarction (STEMI), 1.7% had unstable angina [10]. While sudden cardiac death (SCD) has been reported in 1–5% of cases [11, 33], it remains a rare but serious manifestation. Chest pain is the hallmark symptom, present in 82–95% of patients, often accompanied by dyspnea, diaphoresis, or nausea [1, 10]. Unlike obstructive coronary disease, the chest pain in SCAD may be more variable or atypical, leading to delays in diagnosis or misclassification as non-cardiac [2, 10]. Initial troponin can be negative in up to 20% of cases despite underlying myocardial injury. Electrocardiographic findings range from nonspecific changes to frank ST-segment elevations [10]. A high index of suspicion is essential, especially in younger women without traditional cardiovascular risk factors.

### Natural History and Progression

The long-term prognosis of SCAD is generally more favorable than initially believed, especially in patients managed conservatively. Most lesions heal spontaneously through hematoma resorption, endothelial regeneration, and luminal remodeling, with median healing intervals reported between 5 and 39 months [34, 35]. However, recurrence remains a significant concern. In the Canadian SCAD Registry, the 3-year major adverse cardiovascular event (MACE) rate was 14%, with recurrent myocardial infarction occurring in 10%, revascularization in 3.5%, and mortality as low as 1% [3]. Similar findings were reported in the Australian-New Zealand SCAD cohort, which documented a 3-year MACE rate of 11.3%, including 5.7% recurrent MI, 1.7% revascularization, and 1.5% heart failure [11].

Early angiographic progression within 30 days occurs in 10–15% of patients [10, 22], while long-term recurrence rates range from 5 to 30% over 3 to 10 years [35–37], depending on cohort characteristics and follow-up duration. In some patients, late healing or de novo dissection in previously unaffected vessels may contribute to these events [3, 11, 22]. Persistent or recurrent chest pain is also common among SCAD survivors, even in the absence of new ischemia. Invasive coronary physiology studies suggest that coronary microvascular dysfunction (CMD), vasospasm, or autonomic dysregulation may be responsible for symptoms in many patients [4, 5]. One study found that more than 70% of SCAD patients with post-event angina demonstrated abnormal coronary flow reserve or elevated microcirculatory resistance [38]. This persistent symptom burden often leads to repeat hospital visits and significantly affects quality of life. Many patients report emotional distress, anxiety, depression, or post-traumatic stress disorder (PTSD), highlighting the importance of integrating mental health support into long-term SCAD care [20, 39, 40].

Pregnancy-associated SCAD (P-SCAD) represents a particularly high-risk subgroup. Events most often occur during the third trimester or early postpartum period and are associated with a more severe clinical course. Compared with non-pregnancy SCAD, P-SCAD more frequently involves ST-elevation MI, cardiogenic shock, cardiac arrest, and high-risk anatomy such as left main or multivessel involvement [14, 41, 42]. In one of the largest P-SCAD series, 24% of patients experienced cardiogenic shock, 14% had cardiac arrest, and 4.5% died [2, 42]. European registries suggest that mortality is often due to sudden arrhythmic death [41], underscoring the need for prompt recognition. Expert consensus algorithms have been proposed to guide care in this high-risk group [43].

Another vulnerable cohort includes patients with inherited connective tissue disorders, such as vascular Ehlers-Danlos, Loeys-Dietz, and Marfan syndromes. Although these conditions account for a small proportion of SCAD cases, they are associated with greater arterial fragility and a higher risk of recurrent or extensive dissection. In both populations, early diagnosis, careful imaging surveillance, and individualized management strategies are critical [1, 4, 20].

### Imaging Strategies for SCAD

SCAD presents unique diagnostic challenges, requiring a nuanced imaging approach that integrates both invasive and non-invasive modalities (Table 1). In the acute setting, invasive coronary angiography (ICA) remains the cornerstone of diagnosis. However, its two-dimensional nature and inability to visualize the vessel wall limit its utility in detecting intramural hematomas (IMH) or subtle dissections, particularly in type 2 and type 3 SCAD [9, 44].

**Table 1:** Current and emerging roles advanced multi-modality cardiovascular imaging in SCAD

	Imaging Modality	Current Practice		Future Perspectives
Invasive Imaging Techniques	**Invasive Coronary Angiography (ICA)**	First-line in acute SCAD; identifies angiographic patterns.	Limited in visualizing vessel wall, especially in Type 2 and 3 lesions; Risk of iatrogenic complication.	Integration with AI-assisted recognition of subtle patterns; enhanced diagnostic algorithms.
	**Intravascular Ultrasound (IVUS)**	Identifies vessel wall structure and IMH.	Lower spatial resolution than OCT.	3D imaging; Real-time vessel wall characterization.
	**Optical Coherence Tomography (OCT)**	High-resolution imaging; Exceptional appearance of intimal tears, false lumen, dissection flaps, and IMH.	Lower penetration of the lumen than IVUS. Requires contrast injection with risk	Technological advancements to allow deeper penetration and safer imaging in fragile vessels.
Non-Invasive Imaging Techniques	**Cardiac CT Angiography (CCTA)**	Noninvasive follow-up; Evaluates healing.	Limited role in acute SCAD, may miss distal lesions	Photon-counting detector CT with higher spatial resolution, discomposed different vessel materials, small vessel and subtle vascular changes detection; Detection of pericoronary adipose tissue;
	**Cardiac MRI (CMR)**	Evaluates acute or chronic myocardial injury, LV function, and SCAD-related myocardial tissue characteristic pattern; Assessment of CMD during follow-up; Identifies myocardial infarction patterns and distinguishes SCAD from MINOCA mimics (e.g., myocarditis, Takotsubo	Limited in direct diagnosis of SCAD;	Radiomics for microinfarction and prognostic insights.
	**Whole-body Vascular CT or MR**	Used to assess associated arteriopathies (e.g., FMD, aneurysms).	May miss subtle arteriopathies; variable protocols and limited standardization across centers.	AI-assisted vascular phenotyping and standardized screening.

Abbreviations: AI: artificial intelligence; IMH: intramural hematomas; FMD: Fibromuscular dysplasia

SCAD typically manifests as a single-vessel dissection, most commonly involving the mid-to-distal left anterior descending artery [45–47]. Three primary angiographic patterns, as described by Saw et al. [44], include: Type 1 (pathognomonic with multiple lumens and contrast staining), Type 2 (diffuse smooth narrowing), and Type 3 (focal lesions mimicking atherosclerosis). A fourth proposed pattern, Type 4, refers to total vessel occlusion with upstream tapering [48]. Angiographic findings such as long, linear stenoses in tortuous segments, lack of atherosclerosis, and mid-to-distal artery involvement raise suspicion for SCAD, though diagnostic certainty may require adjunctive imaging [4, 11, 49]. In cases of uncertainty, intracoronary imaging techniques, including OCT and intravascular ultrasound (IVUS), are valuable for confirming SCAD, especially in cases of angiographic ambiguity. OCT offers superior spatial resolution (10–20 μm) and excels in identifying intimal tears, false lumens, and IMH. However, the need for contrast injection and the risk of dissection propagation limits its use in fragile vessels [25]. IVUS, though lower in resolution (~ 150 μm), is safer in unstable dissections and can delineate hematoma extent without contrast [1].

Non-invasive imaging, particularly coronary computed tomography angiography (CCTA), plays an expanding role in the diagnosis and follow-up of SCAD. While not recommended as a first-line tool in acute high-risk presentations due to limited resolution in distal vessels, CCTA can visualize key features such as abrupt or tapered luminal narrowing, IMH, dissection flaps, and vessel wall thickening [1, 50].

Four hallmark signs on CCTA suggestive of SCAD include [51]: (1) abrupt luminal stenosis > 50% over a length of 0.5 mm; (2) IMH, with eccentric wall thickening, corresponding to type 2 or 3; (3) tapered luminal stenosis > 50%; (4) linear hypodensity represented dissection flap, consistent with type 1 SCAD. Importantly, CCTA can be used to monitor vessel healing over time. In small series, CCTA has demonstrated resolution of SCAD features within 3–6 months in most patients [52].

Emerging applications of CCTA may also provide novel insights into SCAD pathophysiology and risk stratification. Pericoronary adipose tissue (PCAT), a CCTA-derived marker of perivascular inflammation, has shown potential in differentiating SCAD from atherosclerotic presentations and may help identify patients with persistent inflammatory activity [53]. PCAT changes could serve as a non-invasive imaging biomarker in future efforts to risk stratify or monitor SCAD patients. Additionally, CCTA allows for evaluation of coronary tortuosity and high-risk plaque features that may mimic or overlap with SCAD [54]. Beyond coronary imaging, its ability to assess extra-coronary vasculature supports the screening for FMD and other vascular abnormalities that frequently coexist in SCAD patients [55].

Recent advent of CT scanners with photon-counting detectors (PCD) [56] is a significant evolution of conventional CT. PCD-CT offers higher spatial resolution (0.125–0.2 mm), reduced noise, and spectral imaging capabilities, PCD-CT may enhance the detection of subtle wall abnormalities and IMH in distal vessels, broadening the role of CCTA in both initial diagnosis and longitudinal monitoring in SCAD [57].

Cardiovascular magnetic resonance imaging (CMR) is valuable for diagnosing and prognosticating all myocardial infarction types, despite limited coronary visualization. In SCAD, it clarifies ambiguous angiographic or CCTA findings by assessing myocardial injury and LV function [58]. In Acute SCAD, CMR shows edema, perfusion defects, and extent of myocardial infarction. Post-SCAD chest pain is common with 31.8% of patients with persistent chest pain at 3 years follow-up, and while some patients may experience recurrent SCAD or acute coronary syndromes, many others suffer from chest pain due to coronary microvascular and endothelial dysfunction [59, 60] with non-invasive imaging playing a pivotal role in differentiating recurrent dissection from myocardial ischemia. Quantitative perfusion CMR and Positron Emission Tomography (PET) are vital in detecting CMD, which could explain persistent symptoms in patients’ post-SCAD [20].

In summary, the imaging strategy for SCAD should be individualized based on clinical stability, anatomy, and diagnostic certainty. ICA remains essential in acute cases, with intracoronary imaging reserved for ambiguous findings or confirmation. CCTA is a valuable non-invasive tool for follow-up, recurrence assessment, and extra-coronary vascular screening, particularly as photon-counting technology becomes more widely available. CMR serves as an important adjunct for functional and prognostic evaluation. A multimodality, precision-guided approach to SCAD imaging optimizes diagnostic accuracy and supports safer, more tailored patient care.

### Management of SCAD

Management of SCAD differs substantially from traditional ACS care. Thrombolytics are contraindicated in most SCAD cases due to the risk of extension of dissection or hematoma [2, 4]. While early revascularization is standard in ACS, SCAD favors conservative therapy in stable patients due to high rates of spontaneous healing and procedural risks associated with percutaneous coronary intervention (PCI) [1, 3, 11, 34]. These risks include difficulty wiring the true lumen, dissection extension, abrupt vessel closure, and restenosis [22, 34, 47]. PCI success rates are as low as 64%, with durable success in only 30%, whereas < 2% of conservatively managed patients require later revascularization [2, 45]. Conservative management is associated with lower in-hospital MACE [47] and excellent long-term outcomes [3, 11]. Revascularization is typically reserved for ongoing ischemia, left main or multivessel involvement, or hemodynamic instability [1, 2, 4].

The scientific statements on SCAD from the European Society of Cardiology (ESC) and the American Heart Association (AHA) [1, 2] outlined pharmacologic treatments based on prospective series and national registries. Pharmacologic therapy focuses on symptom relief, secondary prevention, and minimizing recurrence.

Beta-blockers, which reduce arterial shear stress—similar to their protective role in aortic dissection by lowering heart rate and arterial pressure – are the most consistently recommended therapy and may reduce recurrence risk [35], though data are mixed [11]. Statins are not routinely indicated unless coexistent CAD is present. For persistent symptoms, nitrates, calcium channel blockers, and ranolazine may be used [1, 2].

If PCI is performed, DAPT is indicated. However, the conundrum lies in the potential benefit of long-term DAPT for conservatively managed patients. Some advocate for following ACS guidelines of 1 year of DAPT followed by lifelong aspirin therapy. Others avoid or limit DAPT, advising its use for at least 2 to 4 weeks after the SCAD event, followed by low-dose aspirin therapy for 3 to 12 months, aligning with the typical SCAD healing period [4]. While antithrombotic therapy is logical in the presence of intimal tears, most SCAD cases involve arterial wall hemorrhage, suggesting that antiplatelet therapy may be less beneficial. Data from the European DISCO SCAD Registry revealed that at 1-year follow-up, patients on DAPT had higher rates of MACE compared to those on single antiplatelet therapy (SAPT) (DAPT: 18.9% vs. SAPT: 6.0%; HR 2.62, 95% CI 1.22–5.61, *p* = 0.013) [61]. Similarly, recent analyses from the Australian/New Zealand SCAD Registry found that ticagrelor plus aspirin (aHR 2.6, 95% CI 2.1–5.3, *p* = 0.01) was associated with higher risk of recurrence at a median of 21-months follow-up [11], which was later corroborated by a meta-regression analysis (*n* = 2,306, median follow-up of 3.5 years) [62]. These findings support the rationale behind the ongoing Randomized Study of Beta-Blockers and Antiplatelets in Patients With Spontaneous Coronary Artery Dissection (BA-SCAD) [63]. Current consensus supports discontinuation of anticoagulation once SCAD is angiographically confirmed [1, 2].

A recent meta-analysis encompassing 53 studies and 8,456 SCAD patients demonstrated significant variability in SCAD management, reflecting regional differences in clinical practice [64]. Despite the progress in understanding SCAD pathophysiology and treatment options, considerable uncertainty remains regarding optimal and duration of pharmacologic strategies, especially in conservatively managed patients. This underscores the need for large, well-designed randomized trials. A patient-centered, individualized approach remains essential in guiding therapy.

### Exercise and Rehabilitation

Cardiac rehabilitation (CR) is an established secondary prevention strategy for traditional ACS that has been shown to mitigate risk factors, promote a physically active lifestyle, and facilitate psychological support [65, 66]. CR specifically designed for SCAD patients is strongly recommended​​,​​ as studies have reported improvements in exercise capacity along with patient-perceived physical and mental health benefits following rehabilitation [67–69]. Importantly, CR programs tailored to SCAD patients should provide a comprehensive approach, with supervised exercise progression based on unique patient history, and psychosocial strategies to address fear surrounding physical activity and emotional needs during recovery [70].

Clinical recommendations regarding exercise post-SCAD are largely unstandardized due to a lack of comprehensive data [71]. Limited studies, however, suggest that a gradual return to exercise with an emphasis on moderate intensity aerobic exercise and low intensity resistance training provides a low-risk approach that allows patients to avoid concomitant risks associated with a sedentary lifestyle [2, 72]. Specifically, current exercise guidelines recommend aerobic training at a moderate intensity for 30–40 min at least five days/week [72]. A dedicated warm-up period of 8–10 min should occur prior to exercise to avoid sudden increases in heart rate or blood pressure [70]. Extended bouts of high intensity exercise and exercise in extreme conditions (e.g., heat, heavy contact sports) should be avoided [1].

Following reintegration of aerobic exercise, resistance training at a low intensity is also recommended, and emphasis should be placed on adequate breathing and avoidance of Valsalva-type strain [1]. Some experts caution the use of a generalized exercise prescription and/or strict thresholds based on heart rate and blood pressure in patients with SCAD due to variability in medication regimen (e.g., beta blockers or vasodilators) and patient response to exercise [71]. Instead, exercise recommendations should be personalized, taking into account each patient’s baseline aerobic capacity, strength, and overall clinical context [4]. Few studies have examined the impact of isometric exercises or heavy resistance training in patients with SCAD, and as such, these activities should be avoided [5].

Literature suggests that objective data from cardiopulmonary exercise testing in combination with subjective scales of perceived exertion, such as the Borg RPE scale, may provide a safer and more adaptable tool for developing an exercise program, especially during early recovery [70, 73]. Future prospective trials are needed to fully understand the role of exercise in SCAD recovery and further define an evidence-based approach for safe exercise rehabilitation. Until results from these clinical trials become available, focus should remain on structured CR as a resource to understand individualized symptoms and hemodynamic responses to exercise as well as provide well-rounded support for patients with SCAD.

### P-SCAD Management

Management of SCAD in the pregnant or postpartum population generally follows the principles of non-P-SCAD, favoring conservative strategies and minimal procedural exposure [1, 4]. Special attention must be paid to avoid teratogenic therapies and unnecessary radiation. Even though management principles mirror those of non–pregnancy SCAD, the stakes are higher: P-SCAD is associated with more severe clinical features, including higher rates of multivessel involvement, left main dissection, heart failure, and maternal morbidity [20, 41]. The recommendation to avoid future pregnancy following SCAD is often encouraged, though it is based on Level III evidence and remains subject to debate [2, 4, 74]. Emerging data from small series (totaling 78 pregnancies in 59 women) suggest that most post-SCAD pregnancies are tolerated without major complications, particularly among patients with preserved left ventricular function and no cardiopulmonary symptoms. However, recurrence of SCAD during or shortly after pregnancy occurred in approximately 6% of cases, and MACE was reported in up to 12% [74]. Risk stratification remains limited, and predictors of recurrence are unknown. As a result, expert consensus still favors caution [1, 2]. For women who desire pregnancy after SCAD, a multidisciplinary Pregnancy Heart Team should guide preconception counseling and care. Evaluation should include assessment of ventricular function, extracoronary arteriopathy, current symptoms, and medication regimen [1]. Pregnancy should be delayed at least one-year post-SCAD, and delivery should occur in a tertiary care center with cardiac and obstetric expertise [74]. Planned vaginal delivery is preferred unless obstetric indications dictate otherwise [43]. Until more data are available, pregnancy after SCAD should be approached as high-risk, and decisions must be guided by shared decision-making and individualized risk assessment [1, 2].

### Precision Medicine in SCAD

**Fig. 1:**
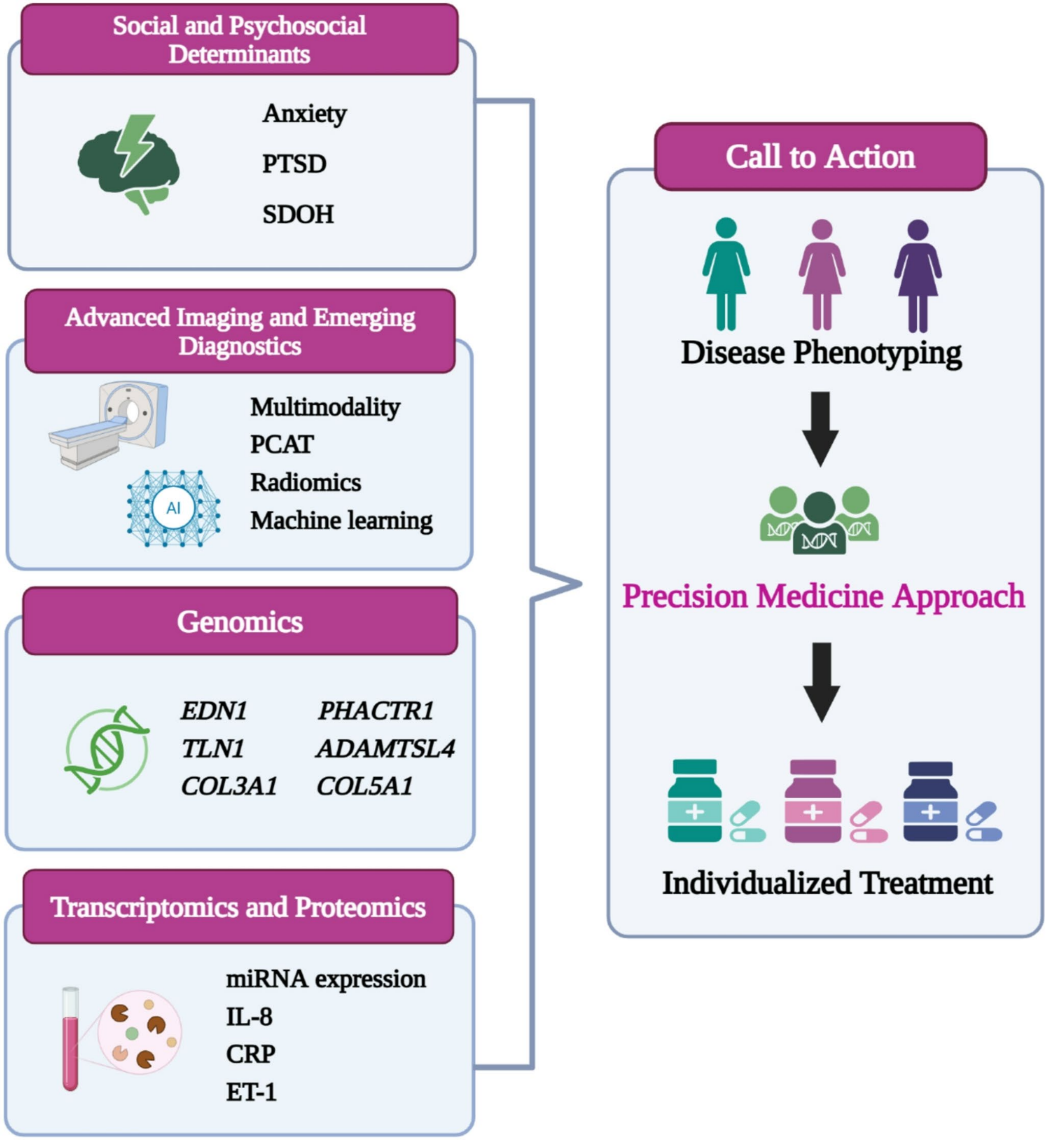
Central illustration toward precision medicine in SCAD: Integrating biological, imaging and social determinants This figure illustrates emerging tools—such as genomics, transcriptomics, proteomics, advanced imaging, and social and psychosocial determinants—that offer an opportunity for SCAD phenotyping and hold promise for improving diagnosis, risk stratification, and tailored treatment. Abbreviations: *ADAMSL4*: a Disintegrin and Metalloproteinase with Thrombospondin Motifs Like 4; *COL3A1*: collagen type III alpha 1 chain; *COL5A1*: collagen type V alpha 1 chain; CRP: C-reactive protein; *EDN1*: endothelin-1 gene; IL: interleukin; ET-1: Endothelin-1 peptide; SDOH: Social determinants of health; PCAT: Pericoronary adipose tissue; *PHACTR1*: phosphatase and actin regulator 1; PTSD: Post-traumatic stress disorder; *TLN1*: Talin-1

The field of cardiovascular medicine is rapidly evolving toward precision-guided care, and SCAD is well-positioned to benefit from this shift. Integrating multi-dimensional data, including genomics, radiomics, transcriptomics, proteomics, metabolomics, and advanced imaging, may enable earlier detection, refined risk prediction, and personalized therapeutic strategies. Although most of these approaches remain exploratory in SCAD, their collective potential provides a foundation for building individualized models of care. This section will summarize the advancements and future directions in this area as applied to SCAD. (Fig. 1. **Central Illustration**).

**Radiomics and Imaging-Based Biomarkers.** Radiomics is a novel imaging analysis technique that extracts high-dimensional quantitative features from standard medical imaging, facilitating deeper phenotypic characterization of vascular disease. This technique involves automated extraction of a wide range of imaging data followed by machine learning–based feature selection aimed to develop models for accurate disease quantification and prediction [7]. While radiomics has been applied in coronary artery disease (CAD) to evaluate plaque composition, lesion vulnerability, and predict adverse cardiovascular events [75], its use in SCAD is still hypothetical. To date, there are no published studies specifically applying radiomics to SCAD cohorts. Nonetheless, the concept is biologically plausible. Radiomic texture and shape features applied to coronary CT angiography may eventually help detect subtle coronary wall heterogeneity or structural vulnerability relevant to SCAD. Similarly, PCAT, assessed via coronary CT, has emerged as an imaging biomarker of vascular inflammation. In CAD, elevated PCAT values have been associated with inflammatory activity and plaque destabilization [76, 77]. Although not traditionally categorized as an omic, PCAT analysis could offer a surrogate measure of vascular inflammation in SCAD and may complement future risk stratification tools [53]. Radiomics applied to PCAT may further enhance its predictive value by quantifying spatial variation in fat texture and density.

While not traditionally classified as an omic, cardiac magnetic resonance (CMR) radiomics represents a promising frontier in myocardial tissue characterization. In MINOCA and other myocardial injury syndromes, pixel-wise texture analysis of CMR images has revealed subclinical fibrosis, edema, and altered tissue architecture [78, 79]. Extrapolating this to SCAD, radiomics may eventually help detect microstructural myocardial changes in dissected territories—such as subtle infarction, inflammation, or remodeling—that are not captured by conventional late gadolinium enhancement. These signatures could ultimately inform prognosis or guide therapy decisions, though no SCAD-specific studies have yet validated this approach.

**Genomics and Heritability.** Multiple genome-wide association studies (GWAS) and candidate gene studies have identified key genetic contributors to SCAD, establishing its heritability. These findings converge on biological pathways involving vascular integrity, extracellular matrix (ECM) organization, endothelial function, and cell-matrix adhesion. Notably, a recent GWAS meta-analysis by Adlam et al. [80] identified 16 risk loci associated with SCAD, including genes implicated in vascular homeostasis and structural integrity. The *PHACTR1/EDN1* locus on chromosome 6 is the most consistently replicated genetic association in SCAD. *PHACTR1* encodes a phosphatase and actin regulator involved in vascular homeostasis, while *EDN1* encodes endothelin-1, a potent vasoconstrictor and inflammatory mediator [81]. The major allele at this locus is associated with increased SCAD risk as well as FMD, migraine, and cervical artery dissection, whereas the minor allele is linked to atherosclerosis and myocardial infarction [82]. Functional studies suggest that *PHACTR1* may regulate *EDN1* expression via enhancer-promoter interactions, though the exact causal mechanism in SCAD remains unclear [82]. Circulating endothelin-1 (ET-1) levels have been associated with cardiovascular risk and inflammation, suggesting a role in vascular instability [81]. The combined influence of this locus is estimated to increase SCAD risk by approximately 70% in carriers of the major allele [82], though further studies are needed to elucidate its pathobiological role.

Other adhesion- and cytoskeleton-related genes have also been implicated. *TLN1* (Talin-1), involved in integrin-mediated cell adhesion and mechano-transduction, harbors rare missense variants enriched in SCAD patients [83, 84]. *F11R (JAM-A)*, which regulates tight junctions and platelet adhesion, has been linked to endothelial dysfunction and thrombosis in SCAD cohorts [85, 86]. Similarly, *ADAMTSL4*, a gene involved in ECM microfibril assembly, has emerged as a candidate susceptibility locus contributing to arterial fragility [87].

Variants in *COL3A1* and *COL5A1*, key genes in collagen synthesis and connective tissue integrity, suggest an overlap between SCAD and vascular connective tissue disorders such as Ehlers-Danlos syndrome [88, 89].

Altogether, these discoveries support a model of SCAD heritability involving both polygenic risk and rare variants. While current evidence does not support routine genetic testing for all SCAD patients, targeted genetic screening may be appropriate in individuals with a family history or syndromic features. Expanding GWAS and sequencing studies in diverse populations will be essential to refine genetic risk models and uncover new biological mechanisms.

**Emerging Omics: Transcriptomics**,** Proteomics**,** and Metabolomics.** Understanding SCAD will require a comprehensive approach that extends beyond genomics alone. Integrating multi-omics data such as transcriptomics, proteomics, metabolomics, and radiomics could offer a holistic perspective on disease mechanisms and potential therapeutic targets, though these areas have been largely understudied. Assessment of plasma miRNAs has shown some promise in identifying a biomarker profile associated with SCAD [90, 91]. Increased abundance of circulating proteins (e.g., IL-8, ET-1, CRP) in patients with acute SCAD compared to conventional ACS and control further suggest their potential role in SCAD mechanisms and warrant future proteomic study [26, 91]. Notably, Olin et al. [92] identified 37-proteins and multiple plasma lipid sub-fractions associated with FMD risk, which may provide a valuable starting point for proteomic analysis in SCAD, as a significant proportion of SCAD patients present with FMD [3].

Despite its promise, multi-omics research in SCAD remains in early stages. Establishing a unique biomarker profile may provide clinical utility in the diagnosis of SCAD patients as well as the continued monitoring to prevent recurrence.

**Psychosocial Burden and Social Determinants of Health of SCAD**. Beyond biological mechanisms, a growing body of evidence highlights the role of psychosocial stress, mental health, and broader social determinants in shaping the experience and outcomes of SCAD patients. Multiple social determinants of health (SDOH) have been proven to adversely impact cardiovascular health in women. Defined as “the social conditions in which people are born, live, and work,” SDOH span five major domains, including economic stability, education, healthcare access and quality, neighborhood and built environment, and social/community context [93]. Given the intersection between SDOH and cardiovascular outcomes, current guidelines recommend evaluation of SDOH to help inform treatment decisions and improve cardiovascular health [94, 95].

SDOH are major drivers of adverse psychosocial and emotional outcomes, both of which can negatively affect cardiovascular outcomes. Financial strain, low socioeconomic status, smoking, obesity, and poor self-rated health have been linked to higher rates of anxiety and depression after cardiac events [96]. Anxiety and depression increase adverse cardiac events as well as mortality. In a longitudinal study following 170 females after acute MI, mortality was highest in depressed females [97]. Anxiety and depression are also linked with younger age [96]. This is pertinent given SCAD predominantly affects younger women and accounts for a large percentage of acute myocardial infarction in women < 50 years.

While the psychosocial burden of cardiovascular disease has been extensively studied, this also extends to SCAD survivors. As previously discussed, anxiety, depression, stress, and fatigue are frequently reported in SCAD survivors [96, 98]. Murphy et al. [96] compared post-cardiac event mental health conditions in SCAD patients versus patients with atherosclerotic MI. SCAD survivors were more likely than their non-SCAD counterparts to experience anxiety, distress, and depression in the 6 months after their acute event, even after controlling for sex, younger age, socioeconomic disadvantage, and mental health history. Rates of post-traumatic stress disorder (PTSD) are also increased in SCAD. In the iSCAD (International SCAD) Registry (*n* = 859) [40], nearly 35% of met criteria for probable SCAD-induced PTSD, significantly higher than rates of PTSD in the general population and higher than rates of PTSD after traditional ACS. Unfortunately, only 35% of patients with probable SCAD-induced PTSD sought treatment for their symptoms.

Given the high levels of psychosocial and emotional impact of SCAD, it is reasonable for SCAD patients to be evaluated using validated screening tools. Suggestions include the Generalized Anxiety Disorder questionnaire (GAD-7) for evaluation of anxiety, Patient Health Questionnaire (PHQ-9) for evaluation of depressive symptoms, and Perceived Stress Scale (PSS) for evaluation of psychological stress.

A qualitative study investigating experiences of SCAD survivors found that “lack of information” was an overarching theme contributing to the adverse psychosocial and emotional impact of SCAD [4, 99]. Although awareness of SCAD has increased over the past several years, the pathogenesis, optimal management and secondary prevention remains uncertain. Continued research on these areas and professional education has the potential to improve the psychosocial and emotional impact of SCAD.

### Individualized Care for Future Precision Medicine

Significant heterogeneity in disease etiology, clinical presentation, and outcomes underscores the need for more precise diagnostic and therapeutic strategies in patients with SCAD. As such, utilizing a comprehensive precision medicine approach, encompassing clinical, molecular, and imaging-based data, may help define distinct SCAD phenotypes and inform tailored interventions.

Although genetic testing is not yet routine in SCAD management, ongoing discovery of risk-associated variants offers promise for future risk stratification and targeted prevention. Identifying individuals with such genetic variants could facilitate preventative lifestyle modifications, targeted pharmacological interventions, and closer surveillance. Additionally, circulating protein biomarkers and inflammatory mediators are also under investigation and may eventually support noninvasive diagnostic algorithms.

Notably, advanced imaging techniques, particularly coronary radiomics are emerging as promising noninvasive tools to capture subtle tissue changes, such as arterial wall heterogeneity or inflammation, that may not be visible through conventional imaging. When integrated with clinical and molecular data, these methods may aid in risk prediction and longitudinal monitoring.

The inclusion of sex-specific differences and SDOH will be critical in advancing personalized care. However, significant knowledge gaps remain in how to best counsel SCAD survivors on hormone replacement therapy (HRT), reproductive care, and future pregnancies. Further exploration of hormonal influences—such as fluctuations during perimenopause, the impact of HRT, and hormonal adaptations during pregnancy—may uncover contributors to vascular vulnerability and SCAD recurrence. Differences in hormonal milieu, vascular structure, and psychosocial context likely contribute to SCAD pathogenesis and outcomes, particularly in women [4, 5]. Future research should prioritize sex as a biological variable and explore how SDOH influence disease expression and therapeutic response.

Current risk stratification tools often fail to identify individuals at risk for SCAD, particularly given its prevalence in younger patients without traditional cardiovascular risk factors [10]. Development of novel risk prediction models, incorporating multi-omic and contextual data, represents a key unmet need in the field.

### Conclusion and Call To Action

SCAD represents a distinct form of acute coronary syndrome, requiring tailored approaches to diagnosis, management, and prevention. Precision medicine—anchored in genomic, proteomic, radiomic, and psychosocial insights—offers a transformative opportunity to improve care for SCAD patients. As deep phenotyping advances, integration of clinical, molecular, and contextual data will be essential to enable earlier identification, stratified management, and ultimately, improved outcomes.

## Data Availability

No datasets were generated or analysed during the current study.

## Key References

2. •• Adlam D, Alfonso F, Maas A, Vrints C, Writing C. European Society of Cardiology, acute cardiovascular care association, SCAD study group: a position paper on spontaneous coronary artery dissection. Eur Heart J. 2018;39(36):3353-68. 10.1093/eurheartj/ehy080.
  - SCAD Expert consensus document by the European Socitety of Cardiology.
3. **••** Saw J, Starovoytov A, Aymong E, Inohara T, Alfadhel M, McAlister C, et al. Canadian Spontaneous Coronary Artery Dissection Cohort Study: 3-Year Outcomes. J Am Coll Cardiol. 2022;80(17):1585-97. doi: 10.1016/j.jacc.2022.08.759.
  - This large prospective, multicenter observational study was conducted across 22 North American centers and followed 750 patients diagnosed with SCAD to investigate the long-term natural history of the disease. The study includes detailed data on baseline demographics, predisposing and precipitating factors, angiographic patterns, treatment strategies, and long- term follow-up for adverse cardiovascular outcomes.
4. •• Hayes SN, Tweet MS, Adlam D, Kim ESH, Gulati R, Price JE, et al. Spontaneous Coronary Artery Dissection: JACC State-of-the-Art Review. J Am Coll Cardiol. 2020;76(8):961 − 84. doi: 10.1016/j.jacc.2020.05.084.
  - A comprehensive review synthesizing recent advances in SCAD, this article updates earlier consensus statements by exploring pathophysiology, clinical presentation, diagnostic strategies, management options, and outcomes, with a focus on individualized and conservative care approaches.
15. •• McAlister C, Alfadhel M, Samuel R, Starovoytov A, Parolis JA, Grewal T, et al. Differences in Demographics and Outcomes Between Men and Women With Spontaneous Coronary Artery Dissection. JACC Cardiovasc Interv. 2022;15(20):2052-61. doi: 10.1016/j.jcin.2022.08.023.
  - Data from the Canadian SCAD Cohort Study and the Non-Atherosclerotic Coronary Artery Disease Registry provide one of the largest comparative analyses of men and women with SCAD. This study offers valuable insights into sex-specific differences in baseline characteristics, clinical presentation, cardiovascular risk profiles, and long-term outcomes.
20. •• Crousillat D, Sarma A, Wood M, Naderi S, Leon K, Gibson CM, et al. Spontaneous Coronary Artery Dissection: Current Knowledge, Research Gaps, and Innovative Research Initiatives: JACC Advances Expert Panel. JACC Adv. 2024;3(12):101385. doi: 10.1016/j.jacadv.2024.101385.
  - This expert panel highlights knowledge gaps in the pathophysiology, diagnosis, and management of SCAD, and outlines future directions to guide targeted research. The authors emphasize the importance of tailored, patient-informed initiatives and introduce the International SCAD (iSCAD) registry as a novel research infrastructure designed to address these unmet needs
50. • Pergola V, Continisio S, Mantovani F, Motta R, Mattesi G, Marrazzo G, et al. Spontaneous coronary artery dissection: the emerging role of coronary computed tomography. Eur Heart J Cardiovasc Imaging. 2023;24(7):839 − 50. doi: 10.1093/ehjci/jead060.
  - A comprehensive review of SCAD emphysized the value of coronary CT angiography as an emerging non-invasive safe imaging modality in SCAD diagnosis and management. CCTA can be effectively used for initial diagnosis and monitoring lesion healing during follow-up
53. • Hedgire S, Baliyan V, Zucker EJ, Bittner DO, Staziaki PV, Takx RAP, et al. Perivascular Epicardial Fat Stranding at Coronary CT Angiography: A Marker of Acute Plaque Rupture and Spontaneous Coronary Artery Dissection. Radiology. 2018;287(3):808 − 15. doi: 10.1148/radiol.2017171568.
  - This study demonstrated that pericoronary adipose tissue (PCAT) analysis on CCTA can identify subtle perivascular epicardial fat stranding, which was also observed in SCAD cases. Incorporating PCAT analysis into CCTA offers a unique diagnostic advantage by detecting culprit lesions and assessing risk beyond traditional lumen-based imaging.
61. •• Cerrato E, Giacobbe F, Quadri G, Macaya F, Bianco M, Mori R, et al. Antiplatelet therapy in patients with conservatively managed spontaneous coronary artery dissection from the multicentre DISCO registry. Eur Heart J. 2021;42(33):3161-71. doi: 10.1093/eurheartj/ehab372.
  - A large, multicenter international registry study from the DIssezioni Spontanee COronariche (DISCO) cohort assessing the role of antiplatelet therapy in patients with SCAD undergoing initial conservative management. This study evaluates patterns of single versus dual antiplatelet therapy (SAPT vs. DAPT) use and explores their association with 1-year clinical outcomes, providing important insights into real-world treatment practices and potential safety considerations in this high-risk population.
70. • Van Iterson EH, Laffin LJ, Svensson LG, Cho L. Individualized exercise prescription and cardiac rehabilitation following a spontaneous coronary artery dissection or aortic dissection. European Heart Journal Open. 2022;2(6):oeac075. doi: 10.1093/ehjopen/oeac075.
  - A clinical practice and education article addressing exercise rehabilitation for acute dissection patients.
80. Adlam D, Berrandou TE, Georges A, Nelson CP, Giannoulatou E, Henry J, et al. Genome-wide association meta-analysis of spontaneous coronary artery dissection identifies risk variants and genes related to artery integrity and tissue-mediated coagulation. Nat Genet. 2023;55(6):964 − 72. doi: 10.1038/s41588-023-01410-1.
  - Meta-analysis involving 1,917 SCAD cases and 9,292 controls identified 16 novel loci associated with the SCAD phenotype. The authors report novel genetic associations and demonstrate high polygenic heritability for SCAD.

## Abbreviations

ACS: Acute coronary syndrome
ADAMTSL4: A Disintegrin and Metalloproteinase with Thrombospondin Motifs Like 4
CCTA: Coronary computed tomography angiography
CMD: Coronary microvascular dysfunction
CMR: Cardiac magnetic resonance imaging
COL3A1: Collagen type III alpha 1 chain
COL5A1: Collagen type V alpha 1 chain
CR: Cardiac rehabilitation
DAPT: Dual antiplatelet therapy
EDN1: Endothelin-1, gene
ET-1: Endothelin-1, peptide
F11R: Junctional adhesion molecule A
FMD: Fibromuscular dysplasia
GWAS: Genome-wide association study
ICA: Invasive coronary angiography
IVUS: Intravascular ultrasound
MACE: Major adverse cardiovascular event
MI: Myocardial infarction
MINOCA: Myocardial infarction with non-obstructive coronary arteries
NSTEMI: Non–ST-elevation myocardial infarction
OCT: Optical coherence tomography
PCAT: Pericoronary adipose tissue
PET: Positron emission tomography
PHACTR1: Phosphatase and actin regulator 1
P-SCAD: Pregnancy-associated spontaneous coronary artery dissection
PTSD: Post-traumatic stress disorder
SCAD: Spontaneous coronary artery dissection
SDOH: Social determinants of health
STEMI: ST-elevation myocardial infarction
TLN1: Talin-1
